# Experimental 3D coherent diffractive imaging from photon-sparse random projections

**DOI:** 10.1107/S2052252519002781

**Published:** 2019-03-20

**Authors:** K. Giewekemeyer, A. Aquila, N.-T. D. Loh, Y. Chushkin, K. S. Shanks, J.T. Weiss, M. W. Tate, H. T. Philipp, S. Stern, P. Vagovic, M. Mehrjoo, C. Teo, M. Barthelmess, F. Zontone, C. Chang, R. C. Tiberio, A. Sakdinawat, G. J. Williams, S. M. Gruner, A. P. Mancuso

**Affiliations:** a European XFEL GmbH, Holzkoppel 4, 22869 Schenefeld, Germany; b Centre for Bio-imaging Sciences, National University of Singapore, 14 Science Drive 4, 117557 Singapore; c Department of Physics, National University of Singapore, 2 Science Drive 3, 117551 Singapore; d Department of Biological Sciences, National University of Singapore, 14 Science Drive 4, 117557 Singapore; e ESRF – The European Synchrotron, 71 avenue des Martyrs, 38000 Grenoble, France; f Laboratory for Atomic and Solid State Physics, Cornell University, Ithaca, NY 14853, USA; g Center for Free-Electron Laser Science, Deutsches Elektronen-Synchrotron, 22607 Hamburg, Germany; h SLAC National Accelerator Laboratory, 2575 Sand Hill Road, Menlo Park, CA 94025, USA; i Stanford Nano Shared Facilities, Stanford University, 348 Via Pueblo, Stanford, CA 94305, USA; j Cornell High Energy Synchrotron Source (CHESS), Cornell University, Ithaca, NY 14853, USA; k Kavli Institute at Cornell for Nanoscale Science, Cornell University, Ithaca, NY 14853, USA; l Department of Chemistry and Physics, La Trobe Institute for Molecular Science, La Trobe University, Melbourne, Victoria 3086, Australia

**Keywords:** coherent X-ray diffractive imaging (CXDI), X-ray free-electron lasers, XFELs, phase problem, single particles

## Abstract

The routine atomic resolution structure determination of single particles is expected to have profound implications for probing structure–function relationships in systems ranging from energy-storage materials to biological molecules. Extremely bright ultrashort-pulse X-ray sources – X-ray free-electron lasers (XFELs) – provide X-rays that can be used to probe ensembles of nearly identical nanoscale particles. When combined with coherent diffractive imaging, these objects can be imaged; however, as the resolution of the images approaches the atomic scale, the measured data are increasingly difficult to obtain and, during an X-ray pulse, the number of photons incident on the 2D detector is much smaller than the number of pixels. This latter concern, the signal ‘sparsity’, materially impedes the application of the method. An experimental analog using a conventional X-ray source is demonstrated and yields signal levels comparable with those expected from single biomolecules illuminated by focused XFEL pulses. The analog experiment provides an invaluable cross check on the fidelity of the reconstructed data that is not available during XFEL experiments. Using these experimental data, it is established that a sparsity of order 1.3 × 10^−3^ photons per pixel per frame can be overcome, lending vital insight to the solution of the atomic resolution XFEL single-particle imaging problem by experimentally demonstrating 3D coherent diffractive imaging from photon-sparse random projections.

## Abstract 2

The application of X-ray coherent diffractive imaging to structure determination of single biomolecules at free electron lasers yields signal levels down to a few hundred scattered photons per frame or less. Here we demonstrate in an analog experiment at a synchrotron source that even at a sparsity level of order 1.3 × 10^−3^ photons per pixel per frame the 3D structure of a nano-fabricated sample can be obtained, even if the orientation of the particle for a given individual frame is not explicitly known.

## 1. Introduction   

A major motivation for advancing coherent diffractive imaging (CDI) using X-rays has always been its potential application to the imaging of individual nanoscale objects. A specific case concerns biological macromolecules, where the structure can be determined without the need for crystallization (Neutze *et al.*, 2000 ▸; Shenoy, 2003 ▸), representing an early potential application of X-ray free-electron lasers (XFELs). The high peak flux, of the order of 10^12^ photons per pulse, and the shorter than 100 fs-scale pulse duration are prerequisites for generating a diffraction signal from a single macromolecule, which is destroyed by the Coulomb explosion of the sample (Neutze *et al.*, 2000 ▸) resulting from ionization during the measurement. This scheme of ‘diffraction before destruction’ is routinely used for serial femtosecond crystallography, in which a randomly oriented crystal is illuminated by the X-ray beam (Chapman *et al.*, 2011 ▸; Schlichting, 2015 ▸). The crystal size has typically been large enough such that sufficient numbers of X-rays are diffracted into a recordable pattern prior to destruction to be able to determine the orientation of the crystal. This is repeated with new microcrystals until there is an adequate sampling of reciprocal space to determine the 3D structure of the unit cell of the crystal.

To obtain 3D structural information from single particles, such as virus particles or single proteins, serial diffraction data from many identical or nearly identical objects has to be measured with sufficient orientational variation. Usually, this is achieved by randomly injecting particles into the FEL beam, relying on the statistical coincidence of a single particle being hit by an FEL pulse (Barty, 2016 ▸; Spence, 2017 ▸). Due to the random nature of this process, the sample’s orientation for each diffraction pattern is generally unknown and has to be recovered *a posteriori* in order to build up a continuous 3D diffraction volume in reciprocal space. This can then be inverted by iterative phase retrieval (Marchesini, 2007 ▸) into a real-space electron-density distribution, the last step of single-particle CDI.

A first complete demonstration of the method was provided by the 3D structure determination of the Giant Mimivirus, approximately 450 nm in diameter, to a resolution of 125 nm (Ekeberg *et al.*, 2015 ▸). More recently, the same type of experiment has been successfully performed using the much smaller Melbourne virus (diameter around 230 nm) to a resolution of 28 nm (Lundholm *et al.*, 2018 ▸). At the same time, a step towards even smaller viruses – the Rice Dwarf Virus (RDV) and bacteriophage PR772, both with a diameter of around 70 nm – has been made, resulting in images at a resolution of around 17 nm (Kurta *et al.*, 2017 ▸) and more recently, for PR772, slighly below 10 nm (Rose *et al.*, 2018 ▸).^1^ Those data were a result of the Single-Particle-Imaging Initiative at the Linac Coherent Light Source (Aquila *et al.*, 2015 ▸; Munke *et al.*, 2016 ▸; Reddy *et al.*, 2017 ▸). This also resulted in the collection of a few hundred high-resolution diffraction frames from RDV at a photon energy of 7 keV, showing that a useful diffraction signal can be collected at 5.9 Å resolution from single hits (Munke *et al.*, 2016 ▸).

Reconstructing biological macromolecules to 3 Å resolution or better has previously been set as the ultimate goal of single-particle CDI (Aquila *et al.*, 2015 ▸). However, when the particle is sufficiently small, only a few hundred X-rays are scattered elastically prior to destruction of the particle (Yoon *et al.*, 2016 ▸; Fortmann-Grote *et al.*, 2017 ▸). In this case the recorded scattering pattern contains too few X-rays to be able to determine the orientation of the particle from the single pattern alone. Such patterns are called ‘sparse’, *i.e.* the features necessary to interpret the pattern are totally dominated by Poisson noise. For example, a protein of at least *ca* 10 nm diameter is necessary to scatter, on average, 50 photons outside the central speckle, at a photon energy of 8 keV in a nanoscale FEL focus (see below for further details). In this case, hundreds of thousands of diffraction patterns have to be collected to build up the 3D reciprocal-space intensity, *i.e.* to assemble an invertible data set (Loh & Elser, 2009 ▸). To date, no such experimental data set exists and considerable method development is still required towards the realization of single-particle CDI as an independent method of macromolecular structure determination.

An important branch of this method development addresses the problem of orientation recovery in the case of very weak diffraction which is often not only sparse, but also contaminated by background signal, originating from sources such as the instrument or the particle beam. In recent years, several methods for orientation recovery have been devised (Huldt *et al.*, 2003 ▸; Loh & Elser, 2009 ▸; Fung *et al.*, 2009 ▸; Bortel & Tegze, 2011 ▸; Tegze & Bortel, 2012 ▸; Giannakis *et al.*, 2012 ▸; Yefanov & Vartanyants, 2013 ▸; Kassemeyer *et al.*, 2013 ▸; Zhou *et al.*, 2014 ▸; Donatelli *et al.*, 2015 ▸, 2017 ▸; Flamant *et al.*, 2016 ▸; Nakano *et al.*, 2017 ▸, 2018 ▸; von Ardenne *et al.*, 2018 ▸) and also applied to experimental single-particle FEL (Loh *et al.*, 2010 ▸; Kassemeyer *et al.*, 2013 ▸; Ekeberg *et al.*, 2015 ▸; Kurta *et al.*, 2017 ▸; Lundholm *et al.*, 2018 ▸; von Ardenne *et al.*, 2018 ▸) or similar data (Nakano *et al.*, 2017 ▸).

However, the important case of sparse diffraction from a 3D object has only been solved experimentally in a setting different from the classical CDI problem. For example, one of the methods of orientation recovery, a statistical technique based on expectation maximization, the expand–maximize–compress (EMC) algorithm (Loh & Elser, 2009 ▸; Ayyer *et al.*, 2016 ▸), has been applied successfully to real-space sparse radiographic data in two (Philipp *et al.*, 2012 ▸) and three dimensions (Ayyer *et al.*, 2014 ▸), to sparse crystallographic data limited to one (Wierman *et al.*, 2016 ▸) and two rotation axes (Lan *et al.*, 2017 ▸), and very recently also to synchrotron-based serial protein crystallographic data for random crystal orientations (Lan *et al.*, 2018 ▸).

Here, we demonstrate 3D CDI from sparse random projections in the same geometry as that used for FEL-based single-particle imaging experiments. Using a synchrotron beam on a micron-scale sample, we show that, with as few as about 50 scattered photons per diffraction pattern, and without explicit knowledge of the sample’s orientation for a given data frame, it is possible to robustly reconstruct the scattering distribution of the sample in reciprocal space and to invert this diffraction volume into a high-resolution 3D electron density distribution. We show that, despite strong sparsity in the data, it is possible to reconstruct a sample to a complexity of more than 40 resolution elements within the largest diameter of the sample. For ease of comparison, the term ‘resolution element’ is defined by analogy with the work of Loh & Elser (2009 ▸) and Ayyer *et al.* (2016 ▸), *i.e.* two resolution elements equal the full-period (crystallographic) resolution. While in simulations it is often defined by the edge of the detector, we define it here in terms of the resolution as obtained after phasing.

## 2. Experimental   

**Figure 1:**
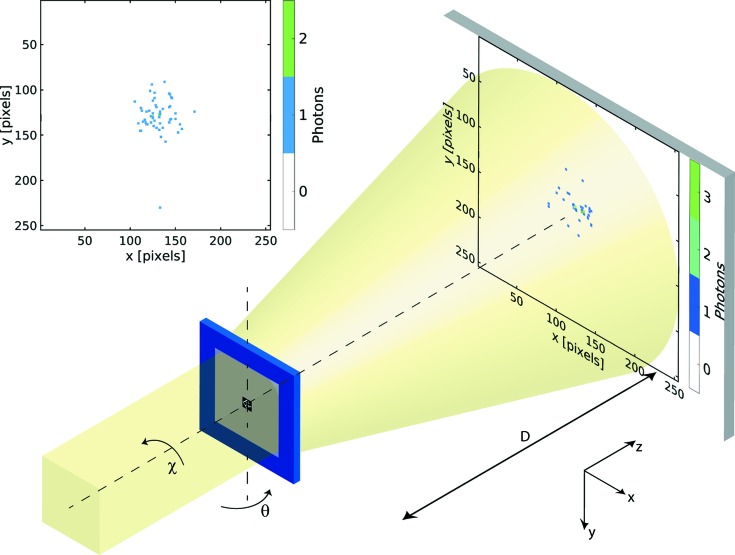
A schematic diagram of the experiment. The sample, a gold nanostructure supported on a silicon nitride membrane, was rotated about the *y* axis by an angle θ to obtain diffraction patterns at different orientations with respect to the optical axis *z*. A first rotation series about the *y* axis was followed by an in-plane rotation of the sample about *z* (angle χ) and a subsequent second rotation series about *y*. The beam attenuation and illumination time were adjusted so that each data frame contains only about 50 scattered photons. An example of a single diffraction pattern is shown in the inset on the upper left-hand side (see also animation in the supporting information). The area of a single pixel in comparison with the field-of-view area has been enlarged for visualization.

The experiment was performed on the undulator beamline ID10, end station EH2, of the European Synchrotron Radiation Facility (ESRF) (Chushkin *et al.*, 2014 ▸). The photon energy was set to 8.1 keV using a water-cooled Si(111) pseudo-channel-cut monochromator with an intrinsic energy resolution of Δ*E*/*E* ≃ 1.4 × 10^−4^. The sample was a solid gold object with a largest diagonal length of about 1.1 μm, fabricated by electroplating and supported by a silicon nitride membrane. It was placed in the beam on a high-precision tomographic stage at a distance of 4.0 m from the detector. Defined by several sets of slits (Chushkin *et al.*, 2014 ▸), the lateral beam size at the sample plane was approximately 10 × 10 μm. A schematic of the setup is shown in Fig. 1 ▸. For further details of all the major aspects of our data treatment and the algorithms employed in the reciprocal- and real-space reconstruction of our data, we refer the reader to the supporting information.

For data collection, the mixed-mode pixel array detector (MM-PAD) was used. It is a wide dynamic range integrating detector developed at Cornell University that is capable of collecting data at a kilohertz frame rate with a high signal-to-noise ratio (SNR), ranging from one X-ray photon per pixel to a maximum rate exceeding 10^8^ photons per pixel per second at 8 keV photon energy (Tate *et al.*, 2013 ▸). The beam was atten­uated using a polished single-crystal Si attenuator with a transmission of *ca* 0.3. As a result, the overall flux reaching the detector was approximately 10^8^ photons s^−1^.

The data set analyzed here comprises diffraction patterns from 227 unique 3D orientations. With 2000 collected frames per orientation, this amounts to a total of *M*_data_ = 454 000 data frames. Each frame was collected with an illumination time of 25 ms. The orientations were obtained from two independent tomographic series (see Fig. 1 ▸), during each of which only θ, the angle about the tomographic rotation axis *y*, was varied.

## 3. Analysis   

### 3.1. Detector calibration and further data treatment   

As the collected data were very sparse, reliable identification of single- and few-photon events becomes crucial for the analysis which explicitly takes into account the Poissonian nature of the noise in the data (see below). Calibration of the detector data, *i.e.* the transformation of the raw detector output into photon counts, is therefore an essential step of the analysis.

The applied calibration procedure consisted of several steps. A binary mask was used to reject all pixels that were inactive or were within gaps between detector modules. Second, the dark signal was subtracted for each pixel. The gain of the detector was determined from the histogram of pixel values over many frames within a region of interest where the maximum intensity per pixel is only a few photons per frame. The resulting histogram shows discrete peaks corresponding to zero, one, two *etc.* photons per pixel. In terms of analog-to-digital units (ADU), a gain of 11.1 ADU photon^−1^ at 8.1 keV was determined from the spacing of these peaks. This gain, together with the widths of these peaks, yields an SNR of 5.2 for single photons. For the transformation of ADUs into single photons, a threshold energy of *E*_t_ = *E*_γ_ − HWHM_n_ = 0.77*E*_γ_ was used, with the single-photon energy *E*_γ_ and the noise peak half-width at half-maximum HWHM_n_.

With around 40 000 active detector pixels in a region of interest (ROI) of 255 × 255 pixels around the beam center, this leads to a false-positive probability *P*(1|0) ≃ 3 × 10^−5^ or between 1 and 2 events per frame. At the sparsity level of the data and with more than 400 000 frames comprising the complete data set, it was found that cosmic radiation made up a considerable contribution to the total recorded intensity. We therefore applied a threshold-based removal procedure. The sparsity level of the single frames is reflected by an average of 49.3 scattered photons detected per frame (median 48 and standard deviation 9.6), after masking out pixels dominated by empty-beam scattering. Due to the low level of background scattering from the instrument, *e.g.* optical components or apertures, no background or ‘empty-beam’ subtraction was performed on the data.

### 3.2. Orientation determination   

The goal of orientation determination is to obtain the 3D reciprocal-space intensity *W*(**q**) that is proportional to the modulus-squared Fourier transform of the 3D electron density of the sample. Here, **q** denotes the 3D Cartesian reciprocal-space coordinate. To reconstruct *W*(**q**), the EMC algorithm correlates each data frame, *K*_*d*_ (*d* = 1, …, *M*_data_), with tomographic slices *W*_*j*_ (*j* = 1, …, *M*_rot_) of *W*, corresponding to *M*_rot_ possible sample orientations, based on the current iterate of our model of *W*(**q**). Each iteration comprises expanding the current model *W* into slices *W*_*j*_, an update *W*_*j*_ → *W*_*j*_′ by maximizing a log-likelihood function *Q*(*W*′), and compressing slices *W*_*j*_′ into a new 3D model *W*′(**q**). The update itself consists of forming the weighted sum 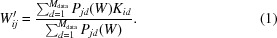
The index *i* specifies a pixel and *P*_*jd*_(*W*) is the probability of a frame *K*_*d*_ having been collected at an orientation *j*, based on the slice *W*_*j*_. For very noisy diffraction data from weak scatterers this probability can be well described using Poisson statistics (Loh & Elser, 2009 ▸).

Each orientation *j* of the sample is represented by a unit quaternion, 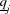. For the present experiment, the set 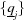 of possible orientations was derived from the experimental setup and procedure (for details, see supporting information). As a result, the optimized set 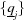 of orientations could be used to generate a Fourier intensity *W*_ref_(**q**), assembled using full knowledge of the orientations, as a reference for the EMC-based intensity reconstruction. EMC received the same set of orientations as an input, together with all data frames, but without explicit knowledge of any frame’s orientation. As a further input, a geometry file was included that contained the reciprocal-space coordinate of each detector pixel and a 3D binary mask, *S*, identifying those voxels in the cubic domain of *W*(**q**) that are to be excluded from the analysis process, *e.g.* because they are never reached by any Ewald sphere slice. As *S* is non-symmetric with respect to **q** = 0, the Friedel symmetrization step included in EMC (Loh & Elser, 2009 ▸) was modified accordingly. To account for the fact that some pixels in each frame *K*_*d*_ contain a large fraction of sample scattering, but still have a significant contribution of ‘parasitic’ or beamline scatter, we defined a binary mask on the detector ROI to identify those pixels to be included in the update rule *W* → *W*′, but not into the calculation of probabilities *P*_*jd*_(*W*) (Ayyer *et al.*, 2016 ▸).

As EMC was always initiated with a random intensity distribution, independent runs of the algorithm show some statistical variation. To reduce the associated uncertainty, the EMC algorithm was run 20 times for 500 iterations, followed by an averaging procedure similar to that described by Yoon *et al.* (2016 ▸). A small fraction of the ensemble, 2 out of 20 reconstructions, exhibited artifacts due to localized over-weighting of certain orientations. These could be automatically discarded by rejecting highly non-homogeneous distributions of orientations. Within the remaining results, two main classes could be observed which are related by an overall rotation of about 180° around an axis close to one of the coordinate axes. This is in accordance with a previous study for an isotropic orientational distribution (Yoon *et al.*, 2016 ▸). After manual attribution to one of the two classes, the results were averaged and their relation was verified by orientational registration. The final averaged 3D reciprocal-space volume 〈*W*(**q**)〉 was obtained as an average of 13 individual results in the same orientation as *W*_ref_(**q**).

## 4. Results   

### 4.1. Reciprocal space (intensity)   

**Figure 2:**
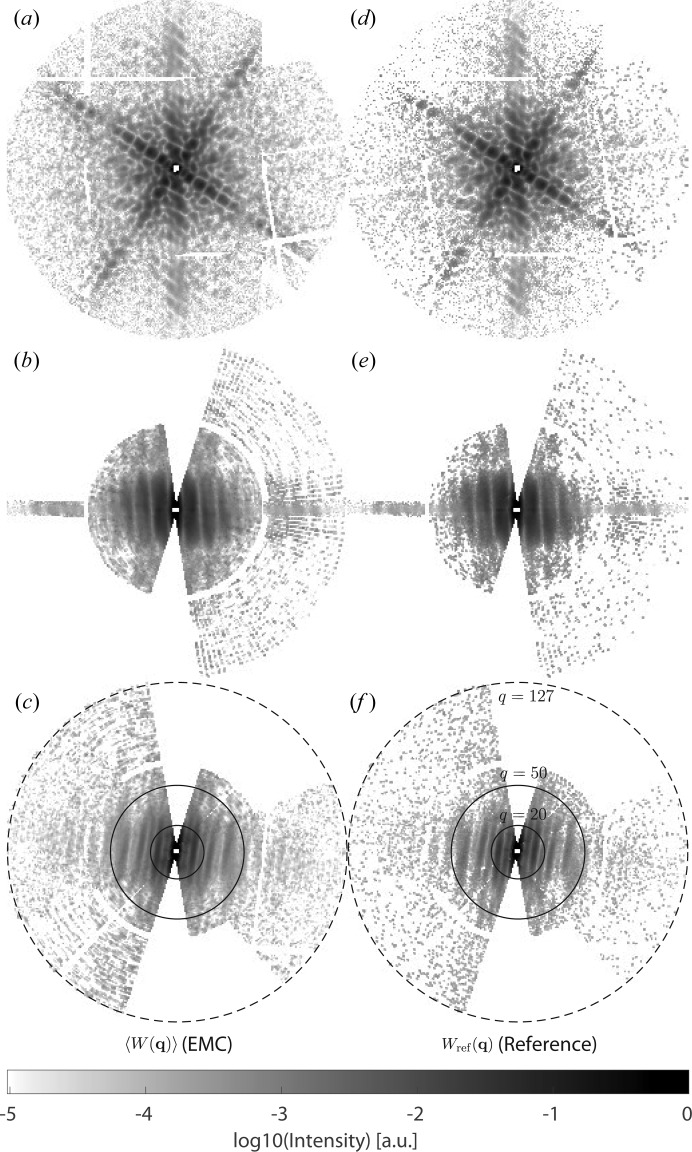
Orthogonal slices through (*a*)–(*c*) the EMC-reconstructed and (*d*)–(*f*) the reference 3D diffraction volumes. The EMC-reconstructed diffraction volume 〈*W*(**q**)〉 results from averaging the results of 13 independent EMC runs, each starting with a random intensity distribution. The reference diffraction volume *W*_ref_(**q**) was constructed based on the known orientations of the sample for each frame during the measurement. The dashed circles in panels (*c*) and (*f*) indicate a radius of 127 voxels, whereas the solid circles indicate radii of 20 and 50, respectively. All slices are drawn on the same scale with dimensionless lateral coordinates in units of Δ*Xk*/*D*.

A comparison of 〈*W*(**q**)〉 with *W*_ref_ is shown in Fig. 2 ▸. Visually, the orthogonal slices through the reconstructed and reference intensities are in very good agreement. This observation is reflected by an overall Pearson correlation coefficient *r* = *C*(*W*_ref_(**q**), 〈*W*(**q**)〉) of 97.1% for **q** ∈ *S* and *r* = 99.0% for 20 ≤ *q* = |**q**| ≤ 50 [see the solid lines in Figs. 2 ▸(*c*) and 2(*f*) ▸].

### 4.2. Validation (intensity reconstruction)   

**Figure 3:**
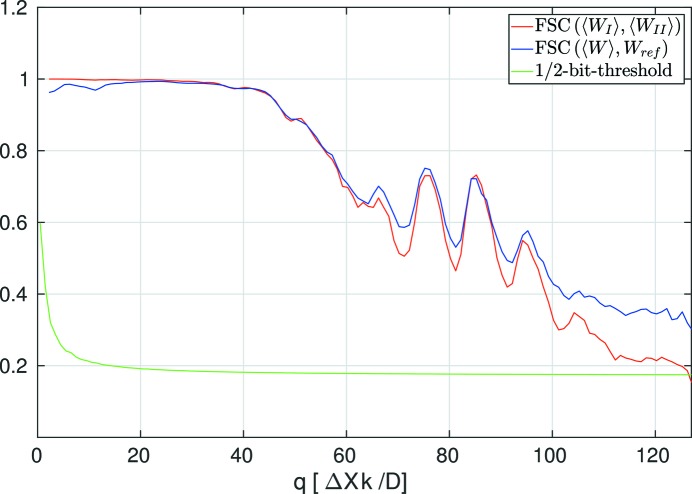
The red line illustrates the Fourier shell correlation (FSC) between two EMC-retrieved reciprocal-space volumes resulting from splitting the data set into two equal halves and performing the same analysis on them as on the whole data set. The blue line indicates the FSC between 〈*W*(**q**)〉 and *W*_ref_, *i.e.* the reciprocal-space volume resulting from analyzing the whole data set using EMC and the reference intensity assembled using known orientations. The green line denotes the half-bit threshold curve, used as a common criterion for resolution determination in analysis of FSC curves.

To assess the reliability of the reconstructed 3D intensity in reciprocal space, we randomly assigned the frames of the data set to two independent half-data sets and reconstructed two independent 3D reciprocal-space volumes as described before. A Fourier shell correlation (FSC) curve (van Heel & Schatz, 2005 ▸), obtained here directly from the two reciprocal-space volumes, is shown in Fig. 3 ▸ (red line). It intersects the half-bit threshold curve (van Heel & Schatz, 2005 ▸) at a value beyond *q* = 120, indicating a self-consistent reconstruction of the reciprocal-space volume close to the Nyquist limit. Here, *q* is measured in units of Δ*Xk*/*D*, where Δ*X* = 150 μm is the detector pixel pitch, *k* is the wave number and *D* is the sample-to-detector distance. (Note that, in the limit of a flat Ewald sphere patch, which is well fulfilled here, *q* then corresponds to the distance to the central beam in units of detector pixels. For further details, see the supporting information.) This analysis shows the effect of EMC alone rather than merging the effects of orientation determination and phasing, as would result for a traditional FSC analysis on phased results in real space. For comparison, the FSC curve resulting from correlating 〈*W*(**q**)〉 with *W*_ref_, as obtained from known orientations, is also shown (blue line). The high degree of similarity between these two curves indicates strong agreement between the result obtained by EMC and the reference intensity distribution, assembled using full knowledge of the orientations.

### 4.3. Real space (density)   

To obtain the real-space electron-density distribution, the missing phases of the 3D Fourier intensity need to be determined. To this end, we applied standard iterative phase retrieval to the 3D diffraction data, *i.e.* a combination of the hybrid input–output (HIO) algorithm and the error reduction (ER) algorithm (Fienup & Wackerman, 1986 ▸; Marchesini *et al.*, 2003 ▸; Xiong *et al.*, 2014 ▸). In total, 600 iterations were applied, *i.e.* 420 iterations of HIO with a feedback parameter β = 0.9, followed by 180 iterations of ER. For further details see the supporting information.

**Figure 4:**
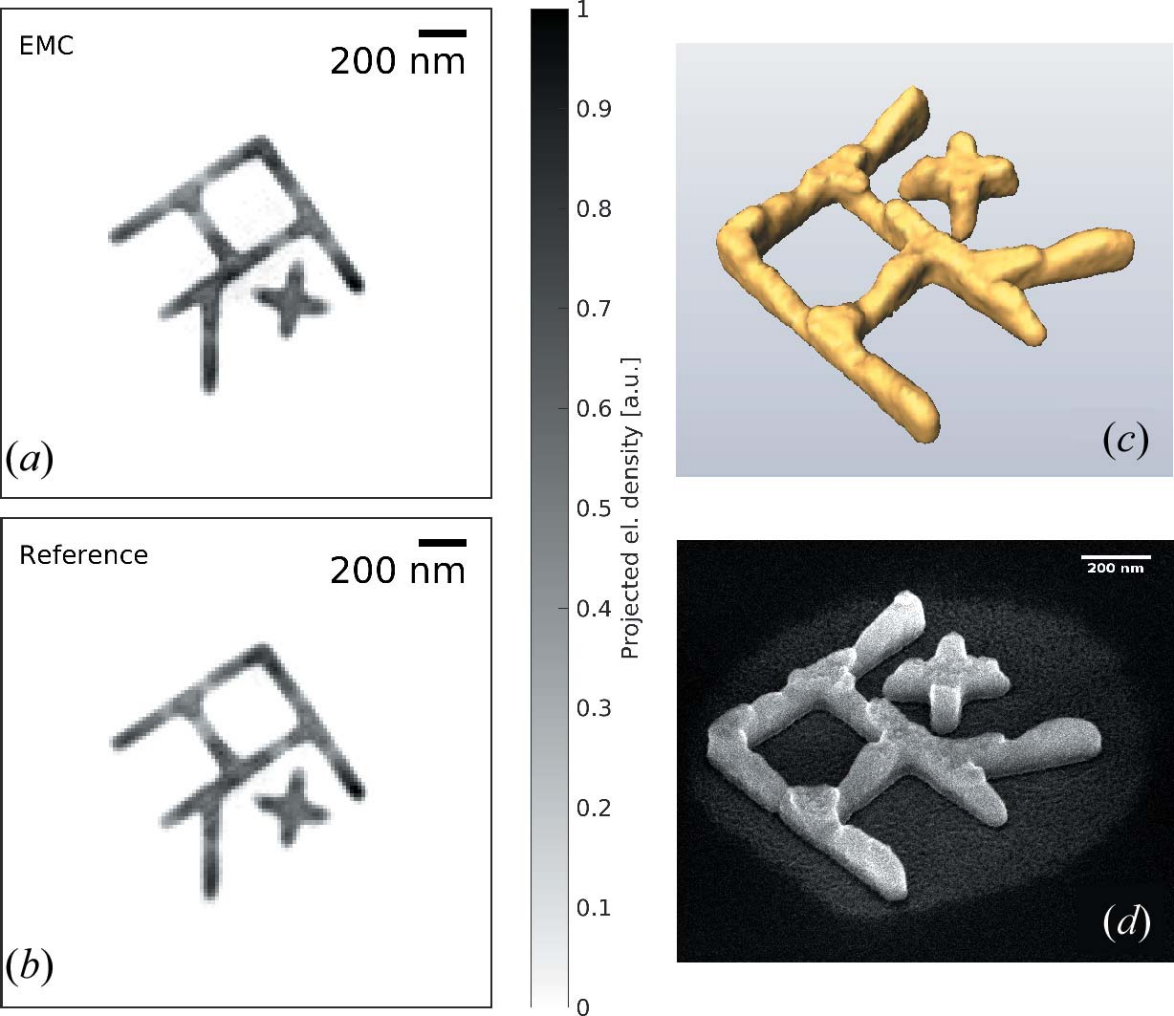
Reconstruction of the 3D electron density. (*a*) Reconstruction from the result derived by EMC. The electron density projected along an axis perpendicular to the drawing plane is shown here. (*b*) Reconstruction from the reference Fourier volume. Again, the projected electron density is shown. (*c*) 3D iso-surface rendering of the reconstructed electron density shown in panel (*a*). The threshold of the iso-surface has been set to 0.2, given a normalized density with values between 0 and 1 (see also animation in the supporting information). (*d*) Scanning electron micrograph from the original sample.

To ensure the reproducibility of the obtained result, 60 reconstructions were performed in total. The results were filtered in a two-step selection process. In the first, manual, step, we discarded images which visually deviated from the most abundant reconstruction result. In the second step, the remaining reconstructions (43 for the reference and 37 for EMC data set) were aligned with sub-pixel precision (Guizar-Sicairos *et al.*, 2008 ▸) and averaged. Then the 20 reconstructions showing the highest correlation with this average were selected for the final average. Note that the procedure applied in the second step could also be used to avoid any manual intervention. However, in such a case, several iterations would likely be required in order to avoid bias by strong outliers in the average. The resulting real-space reconstructions, *i.e.* the real part of the final average, from both reciprocal-space intensities are shown in Fig. 4 ▸.

All details of the reference reconstruction are reproduced in the EMC-based reconstruction down to a resolution level of very few pixels. A comparison of a scanning electron microscopy image of the sample with an iso-surface rendering of the EMC-based reconstruction shows that height variations due to imperfections in the fabrication process are well reproduced by the reconstruction. This identifies the sample as a true 3D structure with features in all coordinate directions.

### 4.4. Validation (density reconstruction)   

**Figure 5:**
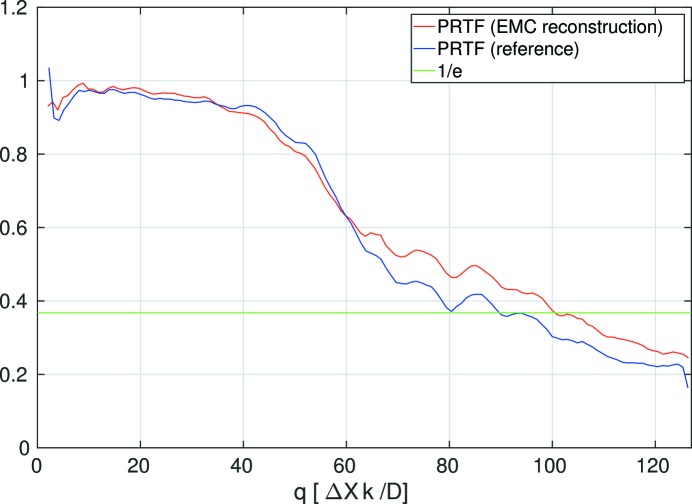
Phase retrieval transfer functions for the reconstructions from the EMC-generated Fourier space intensity 〈*W*(**q**)〉 and the reference intensity *W*_ref_. The curves decay to a value of 1/*e* between *q* = 90 and *q* = 100, corresponding to a half-period resolution between 20 and 23 nm.

The resolution of the final image was estimated via the phase retrieval transfer function (PRTF) according to a procedure similar to the one described by Chapman *et al.* (2006 ▸). More specifically, before summation of the complex-valued reconstructions, their constant phases were adjusted so that the real part of each reconstruction was maximized. The PRTF curves for the results shown in Fig. 4 ▸ are shown in Fig. 5 ▸. The full-period resolution, as determined by the spatial frequency corresponding to a PRTF value of 1/*e*, amounts to a value between 40 and 45 nm. This corresponds to 24 to 26 (full-period) resolution elements within the largest linear extension of the particle, as given by the smallest sphere completely containing the particle (diameter ≃ 1.1 μm).

## 5. Discussion   

### 5.1. Significance of the observed level of sparsity   

To assess the significance of the sparsity level for a single detector frame in the present experiment, we have calculated the expected average total number of scattered photons outside the central speckle for a selection of 35 000 human protein structures from the RCSB Protein Data Bank (PDB; http://www.rcsb.org) (Berman *et al.*, 2000 ▸). Here, a focal-spot diameter of 300 nm was assumed, at a photon energy of 8 keV and a pulse energy of 1 mJ (with 20% beamline transmission). As a result, it could be shown that, under these realistic conditions, the minimum diameter for a protein to scatter 50 photons outside the central speckle amounts to 10.6 nm. For further details, see the supporting information.

This clearly shows that the signal level in the present experiment, obtained at a synchrotron source from a nano-fabricated gold structure, is comparable with what can be expected under realistic conditions from a relevant protein structure at an FEL source.

### 5.2. Particle complexity, rotation group sampling, SNR and sparsity   

Another parameter to be discussed is the particle complexity *R*, as measured in half-resolution units per particle radius (Loh & Elser, 2009 ▸). Reconstructing a particle with 10 nm diameter down to a resolution of 3 Å results in a complexity of *R* ≃ 33, far beyond the current state of the art for FEL-based SPI (Lundholm *et al.*, 2018 ▸) which is *R* ≃ 8 for a globular virus particle. The present structure reaches a complexity >20 in two dimensions, being constrained in the height direction to a value between 2 and 3. A comparison between a scanning electron microscopy (SEM) image and an isosurface rendering of the reconstructed particle density shows that all features in the height direction are very well reproduced. Despite its flat shape, this clearly identifies the particle as a true 3D structure and underlines the significance of the present result as a step forward towards the complexity level required for real protein structures.

Furthermore, compared with a serial imaging experiment at an FEL, where thousands of particles in random 3D orientations contribute to a full data set, the number of unique 3D orientations contributing to the present data set seems relatively low (*M*_rot_ = 227). It can be shown, however, that at the given resolution and complexity this does not restrict the relevance of the result. The required minimum angular separation between adjacent orientations for a sufficient sampling of the 3D rotation group is linked to the complexity *R* of the particle (Loh *et al.*, 2010 ▸): δθ = 1/*R*. In case of a non-globular shape, the maximum complexity in a given coordinate direction allows for a conservative estimate, leading here to δθ = 1/*R*_max_ = 2.2°. This shows that a finer sampling for the tomographic series contributing to the present data set would not have added more information, at the obtained resolution.

Evidently, the present experiment profits from a high SNR (see the supporting information) which would have been impossible without a setup well optimized for forward-scattering CDI (Chushkin *et al.*, 2014 ▸). Most importantly, this consists of a set of accurately placed apertures upstream of the sample which are adjusted to define the beam incident on the sample and, at the same time, to suppress scattering arising from upstream apertures by those further downstream. Similar schemes can be applied at FEL sources to make them compatible with CDI experiments (Munke *et al.*, 2016 ▸). Even though in the FEL case the aerosol jet in which sample particles are injected through a stream of carrier gas causes an additional source of background scatter (Daurer *et al.*, 2017 ▸), the present data set gives an experimental benchmark for a signal-to-noise level which would likely allow FEL-based single-particle imaging.

To provide some understanding of how orientation information can be retrieved despite the sparsity of photon measurements per pattern, consider the two key attributes of a scheme that succeeds in this retrieval. First, this scheme must recognize when it is presented with the correct 3D Fourier intensity distribution, and second, it must contain instructions to improve the compatibility between a candidate intensity distribution and the set of measured diffraction patterns. To satisfy the first attribute of recognizability, the EMC algorithm has to model the detection noise and the randomness of the orientations as accurately as possible. In the present case, this is achieved by a Poissonian noise model and an equal weight for each possible orientation. If a compatible 3D intensity distribution is recognized, then the most likely orientations of each photon pattern within this distribution can be estimated naturally. To fulfill the second attribute, EMC uses this noise model to modify any candidate 3D intensity distribution to increase its likelihood of generating the set of measured diffraction patterns. A derivation of the corresponding maximization rule is given by Loh & Elser (2009 ▸). If there are a sufficient number of photons per pattern, rotation group sampling, signal-to-background ratio and total number of measurements, then EMC can seek and recognize a family of compatible 3D intensity distributions even if the initial guess were completely random (Loh & Elser, 2009 ▸). A more quantitative definition of sufficiency for this orientation problem, beyond what is already in the literature (*e.g.* Loh & Elser, 2009 ▸; Elser, 2009 ▸; Philipp *et al.*, 2012 ▸; Ayyer *et al.*, 2014 ▸; Loh, 2014 ▸), is the subject of a future publication by some of the co-authors here.

## 6. Summary and conclusions   

In summary, we have demonstrated experimentally, in the same geometry as used for FEL-based single-particle imaging, the reconstruction of a complex three-dimensional object using coherent diffractive imaging (CDI) from photon-sparse random projections at a sparsity level to be expected for a typical protein at an FEL source. To this end, we have collected 454 000 data frames with about 50 scattered photons per frame, evenly distributed over 227 unique orientations, and reconstructed a consistent 3D reciprocal-space volume without explicit knowledge of the orientation of a given frame.

It has been shown that, by application of the expand–maximize–compress (EMC) algorithm, both the reconstructed reciprocal-space intensity and the real-space density of the sample agree to a high level with reconstructions obtained using complete knowledge of the frame orientations.

The data set is made freely available in the CXI data bank (Maia, 2012 ▸; http://cxidb.org/), entry 84, to be used as a testbed for algorithm development for CDI-based single-particle imaging, *e.g.* by alternative methods of orientation determination. In addition, the data set can serve as a target for a signal-to-noise level enabling FEL-based single-particle imaging in the future.

## Supplementary Material

Additional details of the experimental and analysis procedures plus supporting figures. DOI: 10.1107/S2052252519002781/it5020sup1.pdf

Iso-surface rendering of the normalized reconstructed electron density, given a threshold value of 0.2. DOI: 10.1107/S2052252519002781/it5020sup2.mpg

A random selection of 100 data frames out of the full data set comprising 454000 frames; note that the pixel size has been enhanced for clarity. DOI: 10.1107/S2052252519002781/it5020sup3.avi

External data: https://doi.org/10.11577/1498641

## Footnotes

1 Unless otherwise noted, we define resolution as the crystallographic (full-period) resolution, in contrast with the half-period resolution which is also often used in the literature on CDI. For comparability, resolution values obtained according to an analysis of the phase retrieval transfer function are given here.
