# Supplementary material for: Experimental 3D coherent diffractive imaging from photon-sparse random projections
Source: IUCrJ. 2019 Mar 20;6(Pt 3):357–65. doi: 10.1107/S2052252519002781 (PMC6503918; doi:10.1107/S2052252519002781)
Supplement: Supplementary file 1 [file m-06-00357-sup1.pdf]

IUCrJ

Volume 6 (2019)

Supporting information for article:

Experimental 3D coherent diffractive imaging from photon-sparse random projections

K. Giewekemeyer, A. Aquila, N.-T. D. Loh, Y. Chushkin, K. S. Shanks, J.T. Weiss, M. W. Tate, H. T. Philipp, S. Stern, P. Vagovic, M. Mehrjoo, C. Teo, M. Barthelmess, F. Zontone, C. Chang, R. C. Tiberio, A. Sakdinawat, G. J. Williams, S. M. Gruner and A. P. Mancuso

Experimental 3D Coherent Diffractive Imaging from photon-sparse random projections: supporting information

K. Giewekemeyer,[1, a)] A. Aquila,[1, b)] N.D. Loh,[2, 3, 4] Y. Chushkin,[5] K.S. Shanks,[6] J.T. Weiss,[6] M.W. Tate,[6] H.T. Philipp,[6] S. Stern,[1, 7, c)] P. Vagovic,[1, 7] M. Mehrjoo,[1, c)] C. Teo,[2, 4] M. Barthelmess,[7] F. Zontone,[5] C. Chang,[8] Richard C. Tiberio,[9] A. Sakdinawat,[8] G.J. Williams,[8, d)] S.M. Gruner,[6, 10, 11] and A.P. Mancuso[1, 12]

[1)] *European XFEL GmbH, Holzkoppel 4, 22869 Schenefeld, Germany*
[2)] *Centre for Bio-imaging Sciences, National University of Singapore, 14 Science Drive 4, 117557 Singapore*
[3)] *Department of Physics, National University of Singapore, 2 Science Drive 3, 117551 Singapore*
[4)] *Department of Biological Sciences, National University of Singapore, 14 Science Drive 4, 117557 Singapore*
[5)] *ESRF - The European Synchrotron, 71 Avenue des Martyrs, 38000 Grenoble, France*
[6)] *Laboratory for Atomic and Solid State Physics, Cornell University, Ithaca (NY) 14853, USA*
[7)] *Center for Free-Electron Laser Science, Deutsches Elektronen-Synchrotron, 22607 Hamburg, Germany*
[8)] *SLAC National Accelerator Laboratory, 2575 Sand Hill Road, Menlo Park (CA) 94025, USA*
[9)] *Stanford Nano Shared Facilities, Stanford University, 348 Via Pueblo, Stanford (CA) 94305, USA*
[10)] *Cornell High Energy Synchrotron Source (CHESS), Cornell University, Ithaca (NY) 14853, USA*
[11)] *Kavli Institute at Cornell for Nanoscale Science, Cornell University, Ithaca (NY) 14853, USA*
[12)] *Department of Chemistry and Physics, La Trobe Institute for Molecular Science, La Trobe University, Melbourne, Victoria, 3086, Australia*

CONTENTS

I. **Experiment** 1

II. **Data analysis** 2
- A. Detector calibration 2
- B. Mapping pixel coordinates to Ewald sphere coordinates 3
- C. Discretization of Fourier and real space 3
- D. Definition of binary detector masks 4
- E. Quaternions and rotation series 4
- F. Alignment of rotation series 5
- G. Friedel symmetrization in EMC 6

III. **Results** 6
- A. Signal versus Background 6
- B. Analysis of retrieved Fourier space results 6
- C. FRC determination 6
- D. Real space reconstruction (phase retrieval) 7

IV. **Summary of essential experimental and analysis parameters** 7

V. **Significance of the observed level of sparsity** 7

References 8

VI. **Supporting tables** 9

VII. **Supporting figures** 10

[a)]klaus.giewekemeyer@xfel.eu
[b)]present address: SLAC National Accelerator Laboratory, 2575 Sand Hill Road, Menlo Park (CA) 94025, USA
[c)]present address: Deutsches Elektronen-Synchrotron, Notkestrae 85, 22607 Hamburg, Germany
[d)]present address: NSLS-II, Brookhaven National Laboratory, P.O. Box 5000, Upton (NY) 11973, USA

I. EXPERIMENT

Beam-defining slits located approximately 0.5 m upstream of the sample were set to a gap of 10 μm in both horizontal and vertical directions resulting in a beam size at the sample position of approximately 10 μm × 10 μm. Diffraction from the beam-defining slits was suppressed by two apertures placed in between the beam-defining slits and the sample[1].

Data collection was initiated with a first rotation series spanning a range of $\theta = -80 \dots 72$ degrees, at one-degree increments. Here, rotations about the coordinate axes by positive angles are defined as left-handed, when looking into the direction of the coordinate axis (see Fig. 1 in the main text). Orientations between $\theta = 46$ and $\theta = 48$ were unintentionally omitted during the process of data colletion which could only be semi-automated to ensure continuous centering of the sample in the beam. Some of the frames collected at $\theta = -16$ were not saved correctly. Therefore, the data for this orientation was excluded from the analysis, so that data from 149 unique

orientations from the first rotation series were used for analysis.

After completion of the first rotation series, the sample was manually removed from the sample stage, rotated by $\chi = -99.37$ degrees about the z-axis, and returned to the sample stage. The latter angle was determined *a posteriori* from the correlation between summed diffraction patterns at $\theta = 0$. The second rotation series consisted of 78 orientations, spanning a range of $\theta = -82 \ldots 72$ degrees, with 2-degree increments.

The ESRF synchrotron was operated in four-bunch-mode, with each bunch carrying around 10 mA of maximum current. With a resulting bunch frequency of 1.42 MHz and with the given attenuation less than 100 photons reached the detector from a single bunch. For a pure counting detector the photons from a single bunch arrive in a time span far too short to be discriminated by the counting electronics. The MM-PAD uses a charge integrating front-end with an extended dynamic range that is achieved by removing a known charge from the pixel input node when the integrator output nears saturation. The number of charge removals is tracked with an in-pixel 18-bit counter. Each charge removal is approximately equivalent to 200 photons at 8.1 keV. The MM-PAD can accommodate an instantaneous x-ray pulse up to this level of 200 photons per pixel per bunch without saturation, allowing, in this experiment, receiving the direct beam on the detector without a central stop. This allowed precise optimization of the beamline settings to suppress parasitic slit scattering and gives the user a large flexibility in selecting software masks to exclude certain detector regions from the subsequent analysis steps, such as those dominated by the central beam.

II. DATA ANALYSIS

A. Detector calibration

The raw signal output for each pixel from the MM-PAD is given in analog-to-digital units (ADUs) which are proportional to the number of electron-hole pairs produced in the Si sensor material of the detector. As further data analysis here requires calibrated detector data, i.e., the number of photons per pixel per frame, the raw signal first needs to be converted accordingly.

Before calibration, a small number of malfunctioning pixels were identified based on their noise level at zero-photon illumination: The root-mean-square (RMS) noise level in each pixel was determined from 15200 dark exposures, equally distributed over the time it took to collect the data analyzed here. Each dark frame was taken with the same exposure settings as the diffraction data frames. A pixel was identified as malfunctioning (and masked out), if its RMS value deviated by more than 10% from the mean RMS value, averaged over all pixels and all patterns. Also, the detector consisted of 6 detector tiles in a 2×3 arrangement. There were gaps between tiles that were insensitive to x-rays and were, therefore, masked out. In all, 6.7% of 105,336 pixels in the whole detector area were masked out for further analysis, including two pixels closest to the beam.

The subsequent calibration process can be described as follows. As a first step, a dark frame was subtracted from each measurement frame. Since dark frames are measurements of the detector output in the absence of signal, and thus subject to the same read noise as other measurements, a common procedure to reduce the noise associated with dark frame subtraction is to define dark frames as an average of many frames, in this case 200. Dark frames are usually static over the short term. Longer term drift is accounted for by periodically updating the subtracted dark frame with new measurements.

Secondly, the gain was determined. To this end, 2000 representative frames from a measurement were used to generate a histogram of raw count rates. As the vast majority of pixels received zero photons during a measurement, a region of interest was defined by selecting those pixels with an average count rate between 5 and 15 ADUs. This defines an interval which is roughly centered around the expected number of ADUs for a single 8.1-keV photon[2].

A Gaussian fit to the left-most (zero-photon, or noise peak) in the histogram resulted in a noise level (standard deviation) of 2.1 ADU. A small offset < 1.0 ADU in the position of the noise peak was determined here as well and corrected for. Subsequently, a Fourier analysis was applied to the histogram to determine the peak separation, yielding a gain of 11.1 ADU for a single 8.1-keV photon. This implies a signal-to-noise (SNR) value of 5.2 at 8.1 keV.

As described previously[3], a threshold E_t can be applied to discriminate single photon events from noise. This step is the central procedure of the detector calibration and is especially relevant, if the data are very sparse, as in the present case. Applying such a threshold inevitably leads to a certain amount of false events, i.e., the detection of a photon where there was none, and vice versa. A natural choice for the threshold parameter is given by the condition that $P(1|0) = P(0|1)$, i.e., the probability of detecting at least one photon when there is none (false positives) equals the probability of detecting no photons, if there is at least one (false negatives). Neglecting multiple-photon events and assuming a Gaussian noise distribution, this condition is given for $E_t = E_\gamma/2$. Note, however, that in this case $P(1|0) = 1/2\mathrm{erfc}(E_t/\sqrt{2}\sigma) \simeq 4.7 \cdot 10^{-3}$ (see Ref. 4). For the present case, with around $40,000$ active detector pixels in the region of interest of a single frame (see below), this would already lead to a false-positive rate of around 180 events per frame. This is far too high, if the expected signal is on the order of 50 photons per frame. A previous study using EMC for reconstruction of real-space tomographic data used $E_t = 0.6E_\gamma$[3]. We have decided here to use $E_t = E_\gamma - \mathrm{HWHM}_n$ where $\mathrm{HWHM}_n = \sqrt{2\ln(2)}\sigma$ is the Half Width at Half Maximum of the noise peak. In numbers, this leads to $E_t \simeq 0.77E_\gamma$, so that $P(1|0) = 3 \cdot 10^{-5}$. As a conse-

quence, the expected number of false-positive events is lowered to less than 2 events per frame. Note that with the chosen threshold we tolerate a false-negative probability $P(0|1) \simeq 0.12$.

As the photon distribution in the data frames is very sparse, cosmic rays are often strongly visible against the low background, especially at high diffraction angles. They usually lead to characteristic streaks several pixels long, with count rates equivalent to a few 8.1-keV photons per pixel. Even though a large body of methods do exist for removing cosmic rays[5], here we utilized the sparse nature of the data to remove them in a simple statistical manner.

Assuming Poisson statistics, the probability for a given pixel i with expectation value λ_i to receive more than a single photon, is given by $P_{\lambda_i}(X \geq 2) = 1 - P_{\lambda_i}(X < 2) = 1 - \exp(-\lambda_i)(\lambda_i + 1)$. To discriminate counts due to cosmic rays from sample diffraction we apply a twofold test on each pixel. First, we determine the expectation value λ_i for pixel i from its mean over all frames. Secondly, if $\lambda_i \leq \lambda_{\text{th}}$ for a threshold expectation value λ_{th}, we regard any count value $X > 1$ as originating from a cosmic ray and set its value to 0. If $\lambda_i > \lambda_{\text{th}}$, we leave the pixel unchanged. The choice of λ_{th} determines the maximum error that we will make during this process, i.e., the maximum number of false identifications per pixel per frame. This includes false deletions of values which originate from sample diffraction (false negatives) and vice versa (false positives). If we accept at maximum one false deletion of a pixel's value per orientation, i.e., per 2000 frames, we can set $P_{\lambda_i=\lambda_{\text{th}}}(X \geq 2) = 1/2000$. As here $\lambda_{\text{th}} << 1$, we can approximate $P_{\lambda_{\text{th}}}(X \geq 2) \approx 1 - (1-\lambda_{\text{th}})(1+\lambda_{\text{th}}) = \lambda_{\text{th}}^2$ and therefore $\lambda_{\text{th}} \approx 1/\sqrt{2000} \approx 0.0224$.

Note that for the majority of pixels λ_i is much smaller than λ_{th}, so that the average number of false positive cosmic ray identifications per orientation is much lower than 1. To determine the average number of false negatives per orientation is more difficult, as this number depends on the expectation value of counts due to cosmic rays, per pixel per frame. In general, the influence of cosmic rays becomes much less relevant as λ_{th} grows. Therefore, we regard their contribution to the total count rate as negligible in this area [1].

B. Mapping pixel coordinates to Ewald sphere coordinates

For a detector (field of view) with a width of N_x pixels and a height of N_y pixels, distributed here on a Cartesian grid, each pixel is represented by a linear index $i = 1, \ldots, N_x \times N_y$ which may be mapped to two-dimensional indices (n_y, n_x) according to

$$n_y(i) = \lceil i/N_y \rceil - L - 1 \qquad \text{(row index)} \qquad (1)$$

$$n_x(i) = (i-1) \bmod N_x - L \qquad \text{(column index)}. \qquad (2)$$

Here, $N_x = N_y = 2L + 1$ with L denoting the distance from the central pixel to the pixel at the edge of the field of view (in pixel units). With this definition, $n_{x,y} \in \{-L, \ldots, L\}$.

In each pixel (n_y, n_x) the diffraction signal at a certain location on the Ewald sphere is measured, whose reciprocal space coordinate is given by Ref. 6

$$q_{(x,y)} = \frac{n_{(x,y)}}{\sqrt{1 + \left(n_x^2 + n_y^2\right)/(L \cot\Theta)^2}} \qquad (3)$$

$$q_z = \frac{L \cdot \cot\Theta}{\sqrt{1 + \left(n_x^2 + n_y^2\right)/(L \cot\Theta)^2}} - L \cdot \cot\Theta, \qquad (4)$$

with $\Theta = \arctan(L \cdot \Delta X/D)$. Here ΔX denotes the pixel pitch of the detector and D the distance of the sample to the detector plane. In this description, the unitless reciprocal space coordinates (q_x, q_y, q_z) are related to their unit-carrying counterparts q'_i via $q_i = q'_i \cdot D/(k \cdot \Delta X)$. Here k denotes the wave number $k = 2\pi/\lambda$ with photon wavelength λ. For simplicity, the coordinate index i will henceforth be omitted.

C. Discretization of Fourier and real space

For compatibility with standard implementations of the Discrete Fourier Transform (DFT) a 3D Cartesian grid is defined in Fourier space with cubic voxels of unit sidelength in dimensionless units, i.e., $\Delta q = \Delta q' \cdot D/(k \cdot \Delta X) = 1$. The grid is defined within a cube of sidelength $M = 2q_{\max} + 1$. A maximum value of

$$q_{\max} = \left\lceil 2D/\Delta X \cdot \sin\left(\frac{1}{2}\arctan\left(\frac{L \cdot \Delta X}{D}\right)\right)\right\rceil \qquad (5)$$

then corresponds to diffraction to the edge of the (region of interest on the) detector. Using this value, it is assured that a circular patch of the Ewald sphere, with a radius corresponding to the distance from the center to the edge of the detector, is always contained within the gridded cube mentioned above. For the present experiment we have chosen L such that $q_{\max} = 127$.

The reciprocity relation of the DFT, $\Delta q' = 2\pi/(M\Delta x')$, gives access to the grid spacing of the corresponding gridded cube in real space, namely $\Delta x' = \lambda D/(M \Delta X)$. Similar to Fourier space, a unitless grid can be defined with lengths measured in units $\Delta x'$ which correspond to DFT-based resolution units defined by the maximum q-vector that is reached by an edge pixel of the detector. Note that these resolution units set a lower (best) limit to the physical resolution of the experiment, but are not necessarily equal to the latter. The dimensionless particle radius R is related to the physical particle radius R' via $R' = R \cdot a$. $a = \Delta x'$ is used in Ref. 7.

---

[1] Another discrimination could be based on a comparison of $\lambda_i^{(\text{diffraction})}$ with $\lambda_i^{(\text{cosmic})}$ for each pixel.

For easier comparison to experimental data, we choose $a = \Delta r$ where Δr corresponds to a half-period resolution element, as obtained from an analysis of reconstructed resolution (see below).

D. Definition of binary detector masks

To optimize the orientation determination using EMC, several binary masks have been defined which describe certain properties for each pixel.

Given a set $M_0 = \{i = 1, \ldots, N_x \times N_y\}$ of pixels with linear index i, the most basic mask of valid pixels is given by the set $M_v \subset M_0$ of all pixels which do not fall on non-sensitive areas between detector modules and are considered as working properly as defined above.

Secondly, a 'beam mask' $M_b \subset M_v$ was defined that excludes all pixels whose signal is dominated by the non-scattered beam or background radiation. More precisely,

$$M_b = M_v \setminus \left\{ i \in M_v \mid \lambda_i^{(\mathrm{sa})}/\lambda_i^{(\mathrm{bg})} < r \wedge \lambda_i^{(\mathrm{bg})} \cdot N_{\mathrm{bg}} > 25 \right\} \tag{6}$$

Here $\lambda_i^{(\mathrm{bg,\ sa})}$ is the mean value of pixel i for background (empty-beam) and sample data, respectively and N_{bg} is the number of frames to define the mean of the background. Consequently, a pixel is considered as dominated by background (including the non-scattered beam), if its signal-to-background value is smaller than r and the mean value of the background itself has been determined with an SNR of at least 5 (Rose-criterion), assuming Poisson statistics. Here we choose $r = 2$ and N_{bg} has a value of 450000 in the present case.

Thirdly, for application of EMC, a mask $M_{EC} \subset M_b$ was defined that includes those pixels to be considered within the expansion and the maximization step of EMC. This mask is defined with respect to the reciprocal space coordinate values $(q_x(i), q_y(i), q_z(i))$ of each pixel on the Ewald sphere, to include only those pixels which correspond to a spherical cap. More precisely,

$$M_{EC} = M_b \setminus \left\{ i \in M_b \mid q_x(i)^2 + q_y(i)^2 + q_z(i)^2 > q_{\max}^2 \right\} \tag{7}$$

with

$$q_{\max} = \left\lceil \max_i \left\{ \sqrt{q_x(i)^2 + q_z(i)^2}, \sqrt{q_y(i)^2 + q_z(i)^2} \right\} \right\rceil . \tag{8}$$

Lastly, a mask $M_M \subset M_{EC}$ of pixels was created which defines the pixels which contribute to the 3D diffraction volume but which are not considered for orientation determination within the maximization step[7]. This allows, for example, to exclude pixels near the center which still have a significant amount of signal from the direct beam which could have a detrimental effect on orientation determination. More specifically, M_M was defined here as

$$M_M = M_{EC} \setminus \left\{ i \in M_{EC} \mid q_x(i)^2 + q_y(i)^2 + q_z(i)^2 < q_{\min}^2 \right\} \tag{9}$$

with $q_{\min} = 7$.

The mask M_M is illustrated in Fig. S1 by white pixels, whereas the difference set $M_{EC} \setminus M_M$ is indicated by gray pixels.

E. Quaternions and rotation series

Following Ref. 8, let $W(\mathbf{q})$ denote the integrated scattering intensity at reciprocal space coordinate $\mathbf{q}$, with the particle fixed in a unique reference orientation. For the given geometry, each detector pixel with index i denotes a unique sampling point $\mathbf{q} = \mathbf{q}_i$ on the Ewald sphere, as described by Eqs. (3) and (4). With the sample in the original reference orientation, $W(\mathbf{q}_i)$ then samples one point of the reciprocal space intensity distribution W.

The goal of the experiment is to homogeneously sample W by changing the relative orientation of the sample with respect to the Ewald sphere. According to Euler's rotation theorem, any orientation of the sample with respect to a given reference orientation can be described by a rotation[2]. Taking the perspective of a fixed sample and a rotating Ewald sphere, the transformation of sampling point $\mathbf{q}_i$ on the Ewald sphere then has to follow the inverse rotation. Thus, if $R_{\mathbf{n}}(\phi)$ denotes the rotation matrix that describes the rotation of the sample (coordinates), then $R_{\mathbf{n}}(\phi)^{-1} = R_{\mathbf{n}}(-\phi)$ describes the corresponding rotation of Ewald sphere coordinates (for a fixed sample). I.e., the new sampling point is given as $W(R_{\mathbf{n}}(-\phi)\mathbf{q}_i)$, or, more generally, $W(R_j^{(ES)}\mathbf{q}_i)$ for orientation j.

EMC implements the determination of a the sample's orientation for a given data frame as a statistical search within a given list of orientations[6]. This list is one of the inputs for the algorithm. For the general case of a freely rotating particle, the possible orientations have to be uniformly spread in the space of 3D rotations (the 3D rotation group $SO(3)$). In the present experiment the free 3D rotation is replaced by two tomographic series (see Section I), with two different, nearly orthogonal rotation axes, so that in total an almost complete coverage of diffraction space is achieved.

Orientation j of the sample during the first series can be described by a rotation matrix $R_j^{(S)} = R_z(\theta_j)$. Here θ_j corresponds to the angles as defined in Section I. The corresponding rotations of the Ewald sphere are thus described by

$$R_j^{(ES)} = [R_j^{(S)}]^{-1} = R_z(-\theta_j). \tag{10}$$

The second series involves sample rotations about two axes (first about z-, then about y-axis), its rotation matrices are given by $R_j^{(S)} = R_y(\theta_j)R_z(\chi)$. The corresponding transformation of the Ewald sphere coordinates is

[2] For $\alpha > 0$ we here define rotations to be counter-clockwise, looking into the direction of $\mathbf{n}$ by angle ϕ about a single axis $\mathbf{n}$.

then represented by the matrix

$$R_j^{(ES)} = [R_j^{(S)}]^{-1} = R_z^{-1}(\chi) R_y^{-1}(\theta_j) = R_z(-\chi) R_y(-\theta_j). \tag{11}$$

EMC uses the quaternion formalism to describe 3D rotations[6]. The quaternion that encodes the rotation by an angle ϕ about an axis $\mathbf{n}$, with respect to a fixed reference orientation, is given by

$$\underline{u}(\phi, \mathbf{n}) = (\cos(\phi/2), \sin(\phi/2)\mathbf{n}). \tag{12}$$

Here $\underline{u}_0 = \cos(\phi/2)$ denotes the 'scalar' part and $\mathbf{u} = \sin(\phi/2)\mathbf{n}$ the 'vector' part of the quaternion. A quaternion norm $\|\underline{u}\| = \sqrt{\underline{u} \cdot \underline{u}}$ can be defined using the standard scalar product $\underline{u} \cdot \underline{v} = \underline{u}_0 \underline{v}_0 + \mathbf{u} \cdot \mathbf{v}$. For counter-clockwise rotations defined as positive, a rotated vector $\mathbf{r}' = \mathbf{R}\mathbf{r}$ may then be obtained by

$$\underline{r}' = \underline{u}^{-1}\, \underline{r}\, \underline{u} =: \mathbf{R}_{\underline{u}}(\underline{r}) \tag{13}$$

with $\underline{r} = (\underline{r}_0, \mathbf{r})$ and $\underline{r}_0 = \underline{r}'_0 = 0$. Here, quaternion multiplication has to be used on the right-hand side of the equation, i.e., $\underline{u}\, \underline{v} = (\underline{u}_0 \underline{v}_0 - \mathbf{u} \cdot \mathbf{v}, \underline{u}_0 \mathbf{v} + \underline{v}_0 \mathbf{u} + \mathbf{u} \times \mathbf{v})$. Upon application of Eq. (13), one may obtain the rotation matrix $\mathbf{R}(\underline{u})$ using elements of $\underline{u}$ (see Ref. 6, Eq. (C1)). Sequential application of Eq. (13) further implies that the application of two rotations in the order $1 \to 2$, can be described by the quaternion product $\underline{u}_1 \underline{u}_2$ (in this order):

$$\mathbf{R}_{\underline{u}_2}(\mathbf{R}_{\underline{u}_1}(\underline{r})) = \mathbf{R}_{\underline{u}_1 \underline{u}_2}(\underline{r}). \tag{14}$$

For the corresponding rotation matrices that act on the vector part of $\underline{r}$ only, we thus have

$$R(\underline{u}_2) R(\underline{u}_1) = R(\underline{u}_1 \underline{u}_2). \tag{15}$$

The first rotation series applied in the present experiment may be described by quaternions $\underline{u}_j(-\theta_j, \mathbf{e}_y)$ (see Eq. (10)). The quaternions $\underline{p}_j$ for the second series may be obtained as a combination of a constant quaternion $\underline{v}$ that describes the new sample orientation at the start of the rotation series, and another quaternion $\underline{u}_j$ that changes for every orientation in the series. With Eqs. (11) and (15):

$$\begin{aligned} R_j^{(ES)} &= R_z(-\chi) R_y(-\theta_j) \\ &= R(\underline{v}(-\chi, \mathbf{e}_z) R(\underline{u}(-\theta_j, \mathbf{e}_y)) \\ &= R(\underline{u}(-\theta_j, \mathbf{e}_y) \underline{v}(-\chi, \mathbf{e}_z)). \end{aligned} \tag{16}$$

Thus, $\underline{p}_j = \underline{u}(-\theta_j, \mathbf{e}_y) \underline{v}(-\chi, \mathbf{e}_z)$. To first approximation (from experimental parameters), $\underline{v}(-\chi, \mathbf{e}_z) = (\cos(-\chi/2), 0, 0, \sin(-\chi/2))$, with $\chi = -99.37$.

F. Alignment of rotation series

If the orientations are known, all $W_{ij} = W(\mathbf{R_j q_i})$ may be interpolated to a 3D diffraction volume $W(\mathbf{p})$ on an equi-spaced Cartesian grid $\mathbf{p}$ in Fourier space, as applied in the compression step of EMC[6]. However, it was observed here that the Fourier space intensities $W_1(\mathbf{p})$ and $W_2(\mathbf{p})$, resulting from the two rotation series do not ideally match, using $\underline{v}$ as defined from the nominal experimental values: By removing the sample frame from its holder, turning it manually about an axis perpendicular to its surface and then returning it to its holder, it is likely that an orientation change slightly different from the expected one has been performed: In the nominal zero-position of the two rotation series the sample frame was likely not exactly perpendicular to the optical axis.

Therefore, we have applied an iterative optimization of $\underline{v}$, based on maximizing the two-point correlation C_{12} between $W_1(\mathbf{p})$ and $W_2(\mathbf{p}, \underline{v})$. Here the argument $\underline{v}$ indicates the dependence of W_2 on $\underline{v}$. This process is equivalent to orientational registration of two scalar fields in three dimensions. For each iteration n, we generated a set of N candidate quaternions

$$V_n = \{\underline{v} \mid d(\underline{v}, \underline{v}_{n-1}) < r_n\} \tag{17}$$

with $\underline{v}_{n-1}$ denoting the best estimate for iteration $(n-1)$. $d(\underline{u}, \underline{v})$ for two unit quaternions $\underline{u}$ and $\underline{v}$ is defined here as

$$d(\underline{u}, \underline{v}) = \min\{2 \arccos(\underline{u} \cdot \underline{v}), 2 \arccos(-\underline{u} \cdot \underline{v})\}, \tag{18}$$

representing a metric on $SO(3)$[9,10] and a pseudometric on $S(3)$, the space of unit quaternions. Note that for $\arccos(\underline{u} \cdot \underline{v}) < \pi$, $d(\underline{u}, \underline{v}) \in [0, \pi]$ is equal to the rotation angle ϕ of the quaternion that maps $\underline{u}$ onto $\underline{v}$ and vice versa. In order to obtain an unbiased set of random quaternions a standard algorithm for uniform sampling of $SO(3)$ was applied[11]. In order to resolve the 2-to-1 mapping from S(3) to SO(3), $\underline{u}$ was replaced with $-u$, if $d(\underline{u}, \underline{v}) < r_n$ for a given $\underline{u}$ and $\arccos(-\underline{u} \cdot \underline{v}) < \arccos(\underline{u} \cdot \underline{v})$. Note that $\underline{u}$ and $-\underline{u}$ define the same rotation, as can be seen by application of Eq. (13).

For each iteration, then the quaternion $\underline{v}_n \in V_n$ was determined which maximizes C_{12} and r_{n+1} was set to $r_{n+1} = d(\underline{v}_n, \underline{v}_{n-1})$. The algorithm was started with $\underline{v}_0 = \underline{v}$ as defined above and $r_1 = \pi/180 \cdot 20$. The algorithm was stopped when $r_n \leq \pi/180 \cdot 1$. To accelerate the calculation of C_{12} only voxels within a shell defined by a radius $R_{\min} = 10 \leq R \leq R_{\max} = 60$ were considered. To speed up the calculation of C_{12} for all candidate quaternions $\underline{v}$, W_2 was not built up from linear interpolation of all Ewald sphere slices for each $\underline{v}$, but it was formed via building up W_2 once for $\underline{v} = 0$ and subsequent rotation of the corresponding Fourier space distribution by a matrix corresponding to the current candidate $\underline{v}$. The latter step was performed using the Matlab routine `imwarp.m`, being part of the Image Processing Toolbox [3].

For the optimized $\underline{v}_{\text{opt}}$ we obtained $d(\underline{v}_{\text{opt}}, \underline{v}) \approx$ 11 deg with respect to the nominal $\underline{v} = (\cos(-\chi/2), 0, 0, \sin(-\chi/2))$, and the rotation axes of $\underline{v}_{\text{opt}}$ and $\underline{v}$ differ by approximately

[3] Matlab R2017b, The MathWorks, Nattick (MA), USA (2017).

$\arccos(\mathbf{v}_{\mathrm{opt}} \cdot \mathbf{v}/(\sin(\arccos(v_0))\sin(\arccos(v_{\mathrm{opt}_0}))) \simeq 7$ deg. After registration, no visual inconsistencies between W_1 and W_2 were observed any more (see Fig. 3 (d-f) of the main text).

G. Friedel symmetrization in EMC

EMC includes an optional Friedel symmetrization step after each iteration, enforcing $W(\mathbf{q}) = W(-\mathbf{q})$ for the current iterate of the 3D Fourier space model $W(\mathbf{q})$[6]. This is often justified due to negligible absorption within the sample. The 3D support mask $S(\mathbf{q})$ identifies all voxels in Fourier space which are reached by a measurement, i.e., an Ewald sphere slice. The set of orientations used in the present experiment defines a support which is not centrosymmetric. Therefore, the standard Friedel symmetrization step in EMC was adapted to the following procedure:

$$W'(\mathbf{q}) = \begin{cases} (W(\mathbf{q}) + W(-\mathbf{q}))/2 & \text{for } \mathbf{q} \in S \wedge -\mathbf{q} \in S \\ W(\mathbf{q}) & \text{for } \mathbf{q} \in S \wedge -\mathbf{q} \notin S. \end{cases} \tag{19}$$

III. RESULTS

A. Signal versus Background

To assess the influence of instrumental background on the data, the azimuthally averaged scattering signal from the sample (including other sources of scatter) and the instrumental background is plotted in Fig. S2. For averaging, the mean signal $\lambda_i^{(\mathrm{bg,sa})}$, as introduced above, has been used. Quite generally, the signal from the sample dominates the background, with a difference in magnitude from about two orders of magnitude at medium-range q down to much less than one order of magnitude at very small and very high q. Here as well as for the calculation of the Fourier Ring correlation plots (see main text) we have used histogram-based azimuthal averaging using the same principle as described in Ref. 12.

B. Analysis of retrieved Fourier space results

Convergence of EMC was monitored using the root-mean-square (RMS) deviation between subsequent iterates of the Fourier space intensity $W(\mathbf{q})$[6]. RMS curves for 20 independent runs of EMC for 500 iterations each are shown in Fig. S3: The majority of runs show a rapid decay of RMS values into a nearly steady state after an initial local maximum. However, there are exceptions, where a distinct local maximum develops at higher iteration numbers.

To arrive at a figure of merit for the quality of the converged result the occupancies N_j of orientations in the last iteration of EMC were investigated. In order to calculate N_j, the most likely orientation for a given data frame as obtained by EMC was used, even though the algorithm itself forms each slice W_j as a sum of all data frames, weighted by their orientational probabilities. Fig. S4 shows the distributions of orientational occupancies N_j, normalized by the constant total number M_j of frames in each orientation ($M_j = 2000$). As the data frames are experimentally equally distributed over all orientations, an equal orientational distribution is the ideal result. For the given dataset, a clear separation between likely and non-likely solutions can be observed, based on the occupancy standard deviation $\sigma(N_j/M_j)$ over orientations j, as shown in Fig. S5(top). In conclusion, all results with a standard deviation $\sigma(N_j/M_j) > 0.2$ were excluded from further analysis. In the present example, these were two out of 20 EMC reconstructions.

In a second step, results were classified according to their overall orientation. To this end, orthogonal slices of the obtained intensity distributions were compared visually with orthogonal slices of the intensity distribution W_{ref}, which had been manually assembled using known orientations of data frames. Two classes could be identified, one of which ('class 1') exhibited strongest similarity with the reference distribution. This visual classification was accompanied by correlating results with a pre-selected reference result. To make this comparison independent of the manually obtained reference distribution which is generally not available in an FEL-based single particle imaging experiment, a representative example of class 1 was chosen as a reference for all other results. Then, the Pearson correlation coefficient was calculated including voxels within a shell ($20 \leq q \leq 50$) in Fourier space where the differences between the two classes were most prominent. The resulting correlation values can also be separated into two groups, based on a threshold of 0.97, as shown in Fig. S5 (bottom).

The resulting intensity distributions corresponding to the two classes of results are illustrated in Fig. S6. Shown here are distributions resulting from averaging over 13 ('Average 1') and 5 ('Average 2') out of 20 intensity distributions. To verify that the relation between the two classes of results is an overall rotation about an axis close to the z-axis, the two Fourier space volumes were registered with respect to each other using the same principle as described in Section II F. After registration, the two distributions exhibit a correlation value of 99.4% within the Fourier space shell $20 \leq q \leq 50$.

C. FRC determination

As described in the main text, the dataset was randomly split into two halves in order to obtain a self-consistent criterion for the validity of the reconstructed reciprocal space volume. EMC was then applied two both half-datasets as described in the previous section. Notably, with only 50% of the frames in each dataset, a significant increase in the number of unsuccessful intensity reconstructions was observed, as indicated by the

standard deviation of occupancy distributions and by artifacts in the reconstructed intensity distributions. From the 20 reconstructions performed for each half 6 were disregarded for the first half and 4 were disregarded for the second half. The applied threshold was the same as before.

The ratio of reconstructions belonging to class 1 and 2 was similar to the whole dataset in both cases (9 vs. 5 and 10 vs. 6, respectively). It is noted that the separation of both orientational classes was not as obvious as for the whole dataset, indicating that M_{data} should not be reduced much below the experimental value for the given dataset.

D. Real space reconstruction (phase retrieval)

To enable convergence, pixels dominated by signal from the direct beam near the center of the 3D diffraction volume were masked out before iterative phasing. As an initial guess for the iterative reconstruction, a sphere with a radius of 40 pixels filled with random values was used. During the 420 HIO iterations the object support was gradually refined using the shrinkwrap algorithm applied at every 20th iteration[13]. Here, an amplitude-threshold of 10% of the maximum was applied to update the support mask, after application of a Gaussian low-pass filter with $\sigma_i = 1/8 + 7/8 \cdot \exp(-i/N_{\mathrm{SW}})$ for iteration number $i = 1, 2, \ldots, 420$ and $N_{\mathrm{SW}} = 420$.

IV. SUMMARY OF ESSENTIAL EXPERIMENTAL AND ANALYSIS PARAMETERS

A summary of essential experimental and analysis parameters of the present experiment is given in Table S1. The values are given here to allow for a detailed comparison to previous (and future) studies on simulated and experimental datasets of Single Particle Coherent Diffractive Imaging. All definitions are in accordance with Ref. 6, unless otherwise noted in the main text. As the sample particle in the present study is non-spherical, its dimensionless particle radius is given here along the direction of the symmetry axes of the smallest rectangular cuboid containing the particle. The linear sampling ratio is given along the same directions. The reduced information rate $r(N) = \langle I(K,W)/I(K,W)|\Omega\rangle$ (see Ref. 6) was averaged here over the last 300 iterations of each run and then averaged over the 13 runs that were used to obtain W_{main} (see main text). $\delta\theta$ defines the minimum angle between two orientations that is at least required to adequately sample the 3D intensity distribution. R_{max} here denotes the radius of the particle in resolution elements along the direction of its largest extension.

V. SIGNIFICANCE OF THE OBSERVED LEVEL OF SPARSITY

The following assumptions were used to estimate the average particle size that elastically scatters a target number of photons (here: 50) per pattern.

1. The number of X-ray photons detected by a detector pixel is assumed to be

$$I(\mathbf{Q}_{\mathrm{pix}}) = I_0 r_e^2 \delta\Omega_{\mathrm{pix}} |F(\mathbf{Q}_{\mathrm{pix}})|^2, \tag{20}$$

   where r_e is the classical electron radius, I_0 is the X-ray fluence, $\delta\Omega_{\mathrm{pix}}$ is the solid angle subtended by the pixel, and $|F(\mathbf{Q}_{\mathrm{pix}})|^2$ is the elastic scattering factor of the particle averaged over all possible particle orientations. $\mathbf{Q}_{\mathrm{pix}}$ here denotes the unit-carrying reciprocal space-coordinate of a detector pixel. We estimate this scattering factor with the Debye scattering equation; here all pixels with a common distance to the central beam on the detector have the same modulus $Q = |\mathbf{Q}_{\mathrm{pix}}|$:

$$|F(Q)|^2 = \sum_{i,j}^{N_{\mathrm{atoms}}} f_i f_j \frac{\sin Q r_{ij}}{q r_{ij}}. \tag{21}$$

   This scattering factor was computed factor for approximately 35,000 human protein structures deposited in the RCSB Protein Data Bank, where the atomic scattering factors (f_i) were taken from Ref. 14. Since we are only targeting resolutions coarser than 5 Å here, the atomic form factors are ignored in these calculations. Further, these proteins were assumed to be in vacuum and not solvated during these scattering simulations.

2. The photon energy has assumed to be 8.1 keV. The focus diameter and pulse energy has been varied within 0.1 to 0.3 μm and 0.1 to 1 mJ, respectively (see Table S2).

3. For simplicity, we simulated photon counts on circular detectors with a fixed maximum resolution of 3 Å, but different beamstops for different proteins such that each beamstop spans the central speckle for each protein structure. We obtain the average total number of elastically scattered photons for each protein by integrating Eqn. (20) azimuthally across the pixels on this detector.

4. The average total photons from the previous step are computed for all 35000 protein structures and scaled to account for different pulse focus diameters. Table S2 shows the diameter of the smallest protein that scatters at least 50 photons across the detector described above.

The number of scattered photons for any selected particle from the PDB, as simulated for the case of 1 mJ pulse energy and 0.3 μm focus diameter is shown in Fig.

S7. It can be seen that hundreds out of the 35000 protein structures scatter more than 50 photons, namely those with a diameter larger than about 10 nm. However, the majority of proteins from the given ensemble has a smaller diameter and scatters even less, under the given conditions.

This shows that the number of scattered photons as observed in the present experiment provides a very realistic test case for an FEL-based SPI experiment from a relevant protein structure.

REFERENCES

[1] Y. Chushkin, F. Zontone, E. Lima, L. De Caro, P. Guardia, L. Manna, and C. Giannini, Journal of Synchrotron Radiation **21**, 594 (2014).

[2] K. Giewekemeyer, H. T. Philipp, R. N. Wilke, A. Aquila, M. Osterhoff, M. W. Tate, K. S. Shanks, A. V. Zozulya, T. Salditt, S. M. Gruner, and A. P. Mancuso, Journal of Synchrotron Radiation **21** (2014), 10.1107/S1600577514013411.

[3] K. Ayyer, H. T. Philipp, M. W. Tate, V. Elser, and S. M. Gruner, Optics Express **22**, 2403 (2014).

[4] J. Becker, D. Greiffenberg, U. Trunk, X. Shi, R. Dinapoli, A. Mozzanica, B. Henrich, B. Schmitt, and H. Graafsma, Nuclear Instruments and Methods in Physics Research Section A: Accelerators, Spectrometers, Detectors and Associated Equipment **694**, 82 (2012).

[5] C. L. Farage and K. A. Pimbblet, Publications of the Astronomical Society of Australia **22**, 249 (2005).

[6] N.-T. D. Loh and V. Elser, Physical Review E **80**, 026705 (2009).

[7] K. Ayyer, T.-Y. Lan, V. Elser, and N. D. Loh, Journal of Applied Crystallography **49**, 1320 (2016).

[8] N. D. Loh, M. J. Bogan, V. Elser, A. Barty, S. Boutet, S. Bajt, J. Hajdu, T. Ekeberg, F. R. N. C. Maia, J. Schulz, M. M. Seibert, B. Iwan, N. Timneanu, S. Marchesini, I. Schlichting, R. L. Shoeman, L. Lomb, M. Frank, M. Liang, and H. N. Chapman, Physical Review Letters **104**, 225501 (2010).

[9] S. M. LaValle, *Planning Algorithms* (Cambrige University Press, 2006).

[10] D. Q. Huynh, Journal of Mathematical Imaging and Vision **35**, 155 (2009).

[11] J. J. Kuffner, in *2004 IEEE International Conference on Robotics and Automation, 2004. Proceedings. ICRA '04*, Vol. 4 (2004) pp. 3993–3998 Vol.4.

[12] J. Kieffer and G. Ashiotis, arXiv:1412.6367 [astro-ph] (2014), arXiv: 1412.6367.

[13] S. Marchesini, H. He, H. N. Chapman, S. P. Hau-Riege, A. Noy, M. R. Howells, U. Weierstall, and J. C. H. Spence, Physical Review B **68**, 140101 (2003).

[14] B. Henke, E. Gullikson, and J. Davis, Atomic Data and Nuclear Data Tables **54**, 181 (1993).

VI. SUPPORTING TABLES

**Parameter**	**Value**
R (particle radius in resolution elements)	ca. $2.5 \ldots 26$, depends on direction
N (mean number of photons per pattern)	49.3
$\tilde{N}$ (median number of photons per pattern)	48
$\sigma(N)$ (standard deviation of photons per pattern)	9.6
$\langle r(N) \rangle$ (mean reduced information rate)	0.86
$\delta\theta = 1/R_{\max}$ (required angular scale of orientations)	2.2°
M_{data} (total number of data frames)	454,000
M_{rot} (total number of unique orientations)	227

TABLE S1. Main parameters of the experiment. As the particle is flat rather than spherical, some parameters vary within a certain range, rather than being constrained to a single value. Minima and maxima here correspond to directions along the particle sides. For further details, see supplemental text.

**pulse energy** / **focus diameter**	1 mJ	0.5 mJ	0.3 mJ	0.1 mJ
0.3 µm	106	148	196	264
0.1 µm	51.2	63.6	74.1	117

TABLE S2. Particle diameter (in Å) of the smallest protein that scatters at least 50 photons per detector pattern when averaged over all orientations, at various pulse energies and focus diameters. This table is computed from more than 35,000 human protein structures in the RCSB Protein DataBank (PDB).

VII. SUPPORTING FIGURES

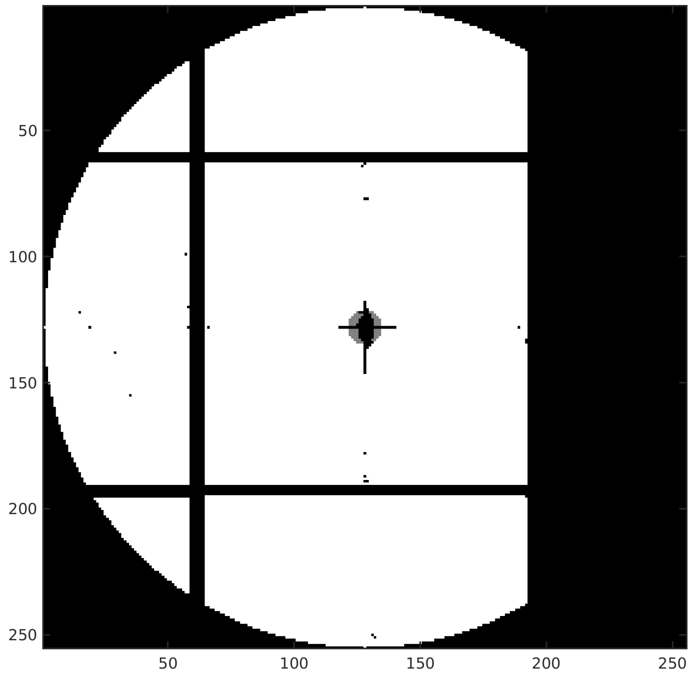

FIG. S1. Detector mask sized 255 by 255 pixels, showing those pixels in white and gray which were used from each frame as an input to EMC. Grey pixels near the center where excluded from orientation determination in the maximization step in EMC, as they still contain mostly non-scattered photons from the primary beam. Axes labels denote pixel numbers.

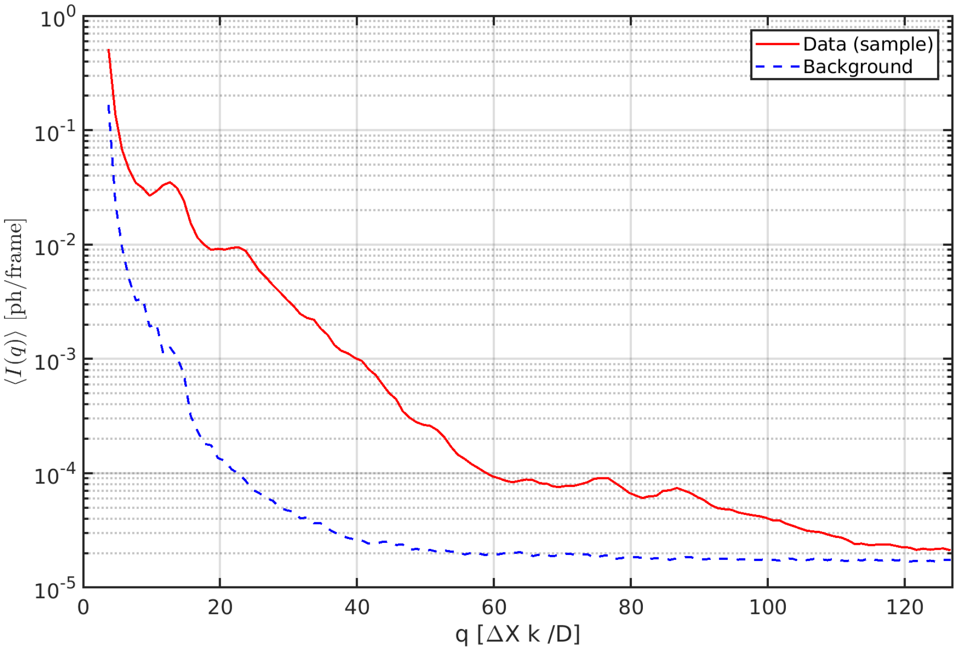

FIG. S2. Azimuthally averaged mean signal from the sample and background versus the dimensionless radial coordinate q (see above and main text).

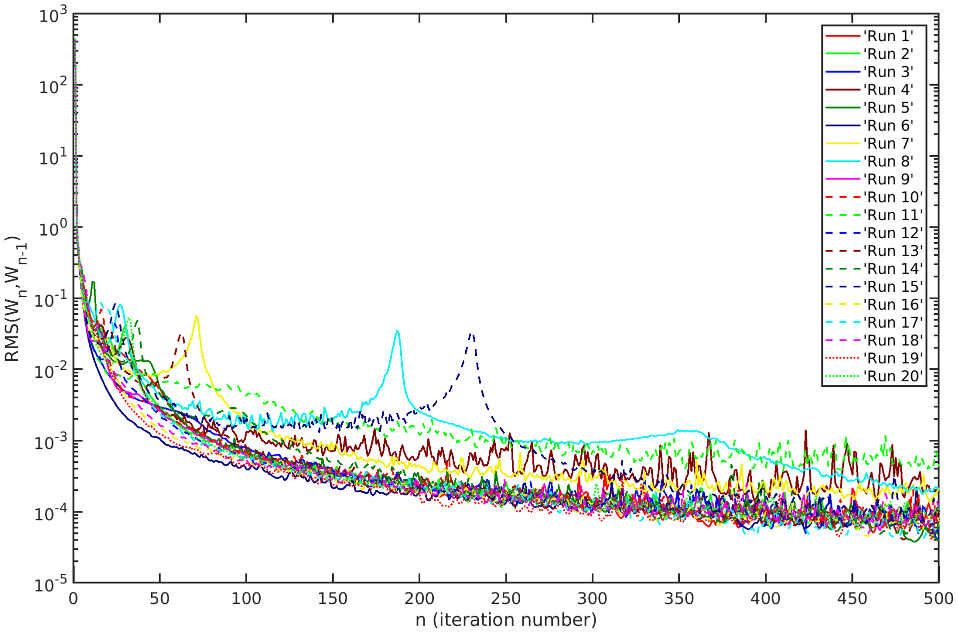

FIG. S3. Root-mean-square change between neighboring iterates of Fourier space intensity $W(\mathbf{q})$ for 20 independent runs of EMC over 500 iterations each.

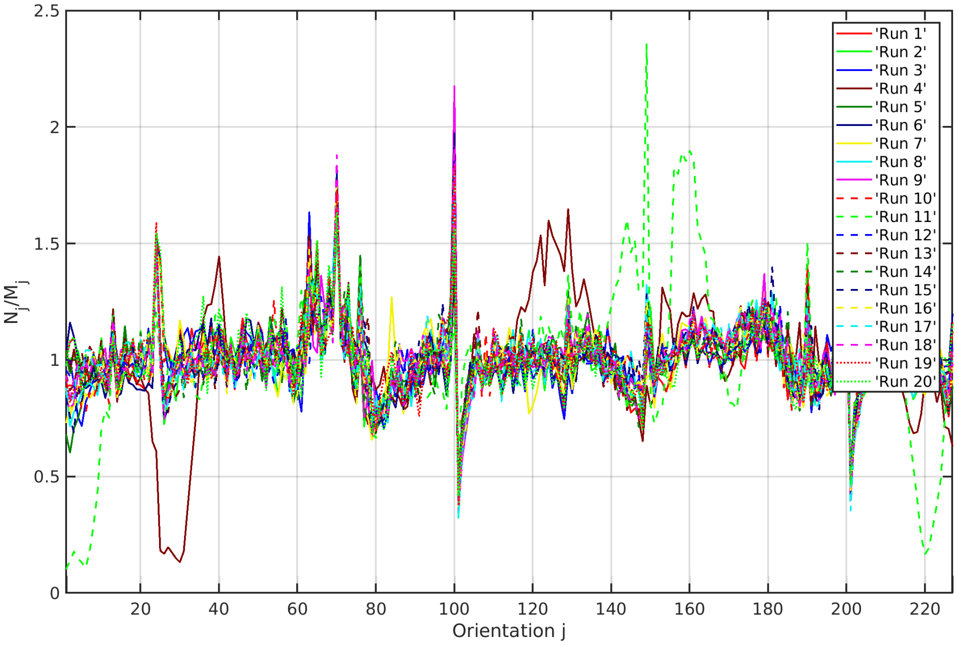

FIG. S4. Distributions of orientational occupancies, normalized by the constant expected occupancy number for each orientation.

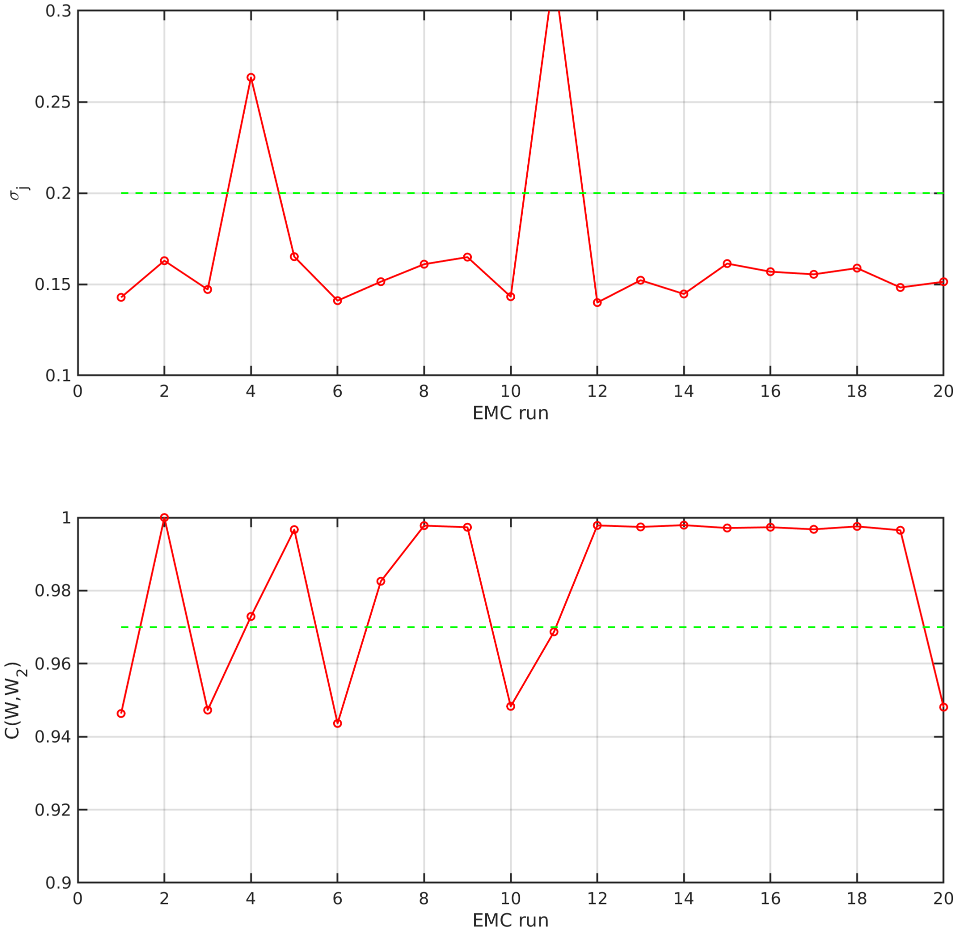

FIG. S5. (top) Standard deviation of normalized occupancy numbers for each run. (bottom) Pearson correlation of obtained Fourier space distributions with a pre-selected reference distribution. Here, the reference distribution was chosen to be the result of run 2.

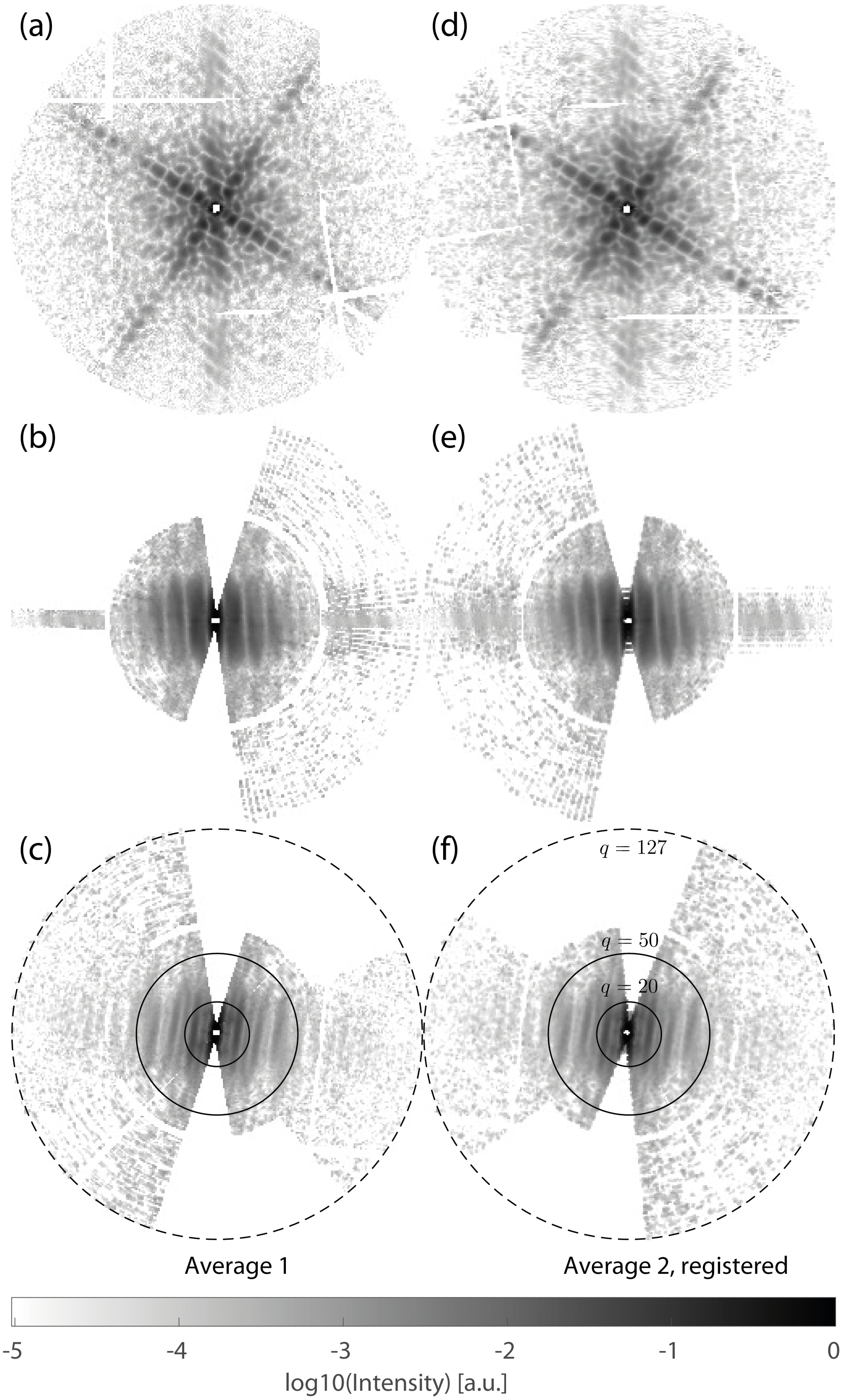

FIG. S6. Orthogonal slices through Fourier space intensities obtained from averaging over two classes of the obtained results (see SM text). The result of class 1 (‘Average 1’; a, b, c), also shown in Fig. 2 of the main text, is compared here to the rotationally registered result of class 2 (‘Average 2’; d, e, f), verifying their close similarity.

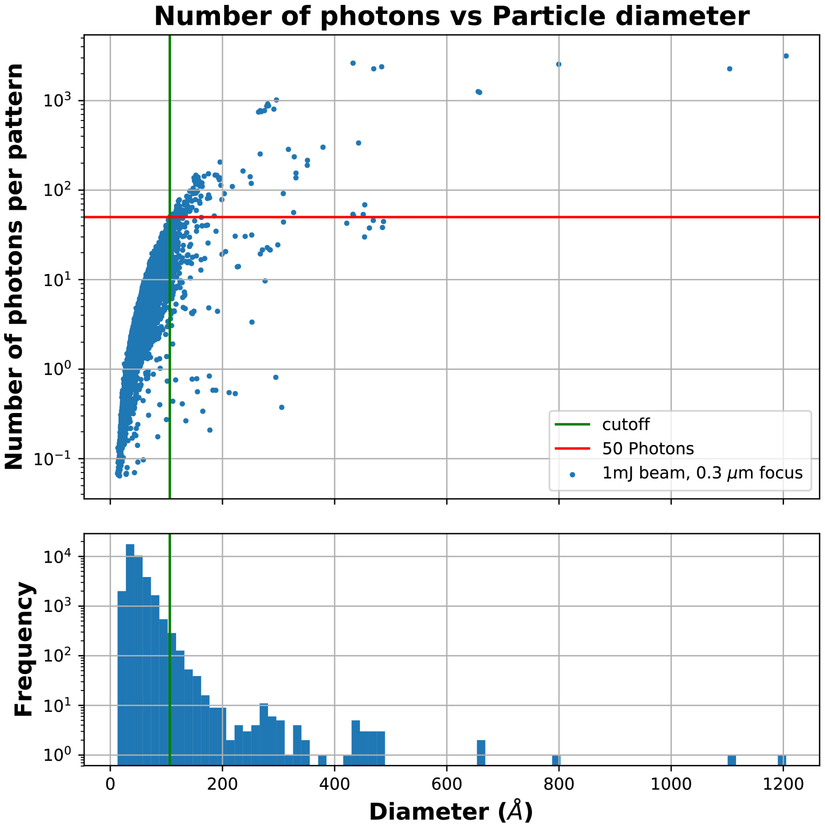

FIG. S7. (Top) The number of scattered photons outside the central speckle is plotted for 35000 human proteins from the PDB against their diameter, assuming an incident pulse energy of 1 mJ and a focus diameter of 300 nm at 8.1 keV photon energy. The red horizontal line indicates a level of 50 scattered photons. The green vertical separates particles which scatter less at given conditions (left) and more (right). The separation is at a particle diameter of 10.6 nm. (Bottom) Size distribution of the 35000 protein structures selected from the PDB.
